# Mobile Health App (AGRIPPA) to Prevent Relapse After Successful Interdisciplinary Treatment for Patients With Chronic Pain: Protocol for a Randomized Controlled Trial

**DOI:** 10.2196/18632

**Published:** 2020-08-18

**Authors:** Stefan Elbers, Jan Pool, Harriët Wittink, Albère Köke, Else Scheffer, Rob Smeets

**Affiliations:** 1 Lifestyle and Health Research Group Healthy and Sustainable Living Research Centre University of Applied Sciences Utrecht Utrecht Netherlands; 2 Department of Rehabilitation Medicine Faculty of Health, Life Sciences and Medicine Maastricht University Maastricht Netherlands; 3 Adelante Centre of Expertise in Rehabilitation and Audiology Hoensbroek Netherlands; 4 South University of Applied Sciences Heerlen Netherlands; 5 CIR Rehabilitation Eindhoven Netherlands

**Keywords:** telemedicine, chronic pain, recurrence, clinical trial protocol, rehabilitation, randomized controlled trial, cost-benefit analysis, treatment adherence and compliance, mobile apps, patient care team

## Abstract

**Background:**

To facilitate adherence to adaptive pain management behaviors after interdisciplinary multimodal pain treatment, we developed a mobile health app (AGRIPPA app) that contains two behavior regulation strategies.

**Objective:**

The aims of this project are (1) to test the effectiveness of the AGRIPPA app on pain disability; (2) to determine the cost-effectiveness; and (3) to explore the levels of engagement and usability of app users.

**Methods:**

We will perform a multicenter randomized controlled trial with two parallel groups. Within the 12-month inclusion period, we plan to recruit 158 adult patients with chronic pain during the initial stage of their interdisciplinary treatment program in one of the 6 participating centers. Participants will be randomly assigned to the standard treatment condition or to the enhanced treatment condition in which they will receive the AGRIPPA app. Patients will be monitored from the start of the treatment program until 12 months posttreatment. In our primary analysis, we will evaluate the difference over time of pain-related disability between the two conditions. Other outcome measures will include health-related quality of life, illness perceptions, pain self-efficacy, app system usage data, productivity loss, and health care expenses.

**Results:**

The study was approved by the local Medical Research Ethics Committee in October 2019. As of March 20, 2020, we have recruited 88 patients.

**Conclusions:**

This study will be the first step in systematically evaluating the effectiveness and efficiency of the AGRIPPA app. After 3 years of development and feasibility testing, this formal evaluation will help determine to what extent the app will influence the maintenance of treatment gains over time. The outcomes of this trial will guide future decisions regarding uptake in clinical practice.

**Trial Registration:**

Netherlands Trial Register NL8076; https://www.trialregister.nl/trial/8076

**International Registered Report Identifier (IRRID):**

DERR1-10.2196/18632

## Introduction

### Background and Rationale

Chronic pain is a major contributor to worldwide disability, affecting approximately 20% of the global population [1-3]. For many patients, ongoing or recurrent pain severely impacts their physical, social, and mental health, as it interrupts ongoing activities and thereby continuously interferes with daily life functioning. Over time, this impacts patients’ sense of self and quality of life [4].

In many cases, there is no monodisciplinary treatment available that can cure the persisting pain. Instead, the multifaceted nature of chronic pain, including biomedical as well as psychological and social factors, often requires a comprehensive treatment approach focusing on improving daily life functioning rather than reducing pain [5-7]. To realize this, interdisciplinary multimodal pain therapy (IMPT) programs have evolved that aim to support patients in learning to live a meaningful life irrespective of pain. These programs share a biopsychosocial orientation toward chronic pain and often include both neuroscientific models of pain physiology as well as (cognitive) behavioral treatment principles [6,8].

Although the effectiveness of IMPT programs has been well established [9-12], maintaining the positive effect of the treatment on patients’ daily lives over time remains a major challenge [13,14]. The problem of relapse is not unique to the domain of pain treatment but has been observed across all health behavior domains (eg, [15,16]). In response, many treatment programs have added relapse prevention strategies that aim to preserve treatment gains over time (eg, [17,18]). In the context of chronic pain treatment, examples of such strategies include self-practice exercises [19,20], booster sessions [21,22], or encouragement of patients to take notes during treatment [23,24]. However, the integration of these particular strategies within the treatment program as well as an underlying theoretical rationale regarding how it may prevent relapse are often not described in clinical studies. Moreover, the effectiveness of these behavior regulation strategies remains unknown because they are usually evaluated as a part of the full program or with a limited follow-up period.

### Mobile Health

The emergence of mobile health (mHealth) provides new opportunities to support behavior regulation to maintain or enhance the long-term treatment effect of IMPT programs. Despite substantial variations concerning study quality, interventions, and outcomes, mHealth apps are generally regarded as a promising strategy to facilitate adherence to treatment principles or to increase self-management skills (eg, [25,26]). A specific advantage is that an app can include multiple interacting behavior regulation strategies within its digital environment (eg, automatically linking personalized goal setting regarding physical activity to accelerometer output). Moreover, mHealth strategies can integrate other smartphone functionalities such as digital calendars, instant messaging services, or a camera, thereby offering personalized behavior regulation strategies that support the transfer of treatment insights into each patient’s personal environment.

Despite the potential and current popularity of mHealth apps, the effectiveness on health-related outcome measures varies greatly [27]. Factors such as engagement—defined as the extent of app use as well as the corresponding subjective experience [28]—and usability—defined as the relative ease with which users can use an app to achieve a particular goal [29,30]—may account for this variability [27,30]. For example, patients that use an app to change their health behaviors may use the app in a different way than intended or stop using the app after several days, which prevents facilitating the intended behavior change [28]. Therefore, evaluations concerning the effectiveness and clinical importance of mHealth apps on health outcomes should take evaluations on user engagement and perceived usability into account [30,31].

### Previous Studies

In 2015, we initiated the SOLACE research project to develop strategies to prevent relapse after IMPT programs. Because there was little research available on this topic [13,14], we started with an 18-month co-design project, in which patients, health care providers, researchers, and designers shared their expertise and collaborated to develop ideas, concepts, and strategies to prevent relapse after successful treatment. This resulted in a prototype paper workbook that contained the two most promising strategies: a valued-based goal-setting procedure, and a method for storing and facilitating retrieval of meaningful treatment experiences. Subsequently, we performed a feasibility study in which the prototype workbook was tested at two different IMPT programs for 6 months. Overall, patients and health care providers were willing and able to use the workbook and regarded the strategies to be in line with the IMPT treatment principles (see personal communication, first author SE; manuscript under review). The evaluations also yielded specific suggestions for further improvements, including a preference for a mobile app instead of a paper workbook, along with more interaction between both strategies and a modified goal-setting procedure. In the ensuing research project (ie, the AGRIPPA project), we used the insights of the feasibility study to improve both strategies and transferred the content of the workbook prototype to an mHealth app. Similar to the initial co-design project, patients, health care providers, designers, and researchers collaborated to optimize usability and intervention components of the app. For example, we explored direct user experience through think-aloud sessions with digital mockups, and we organized cocreation sessions to prioritize app features and to prepare a list of requirements.

### Study Objectives

The present trial has three main objectives. Our first objective is to evaluate the effectiveness of the AGRIPPA intervention (ie, enhanced treatment condition) on pain disability for patients with chronic musculoskeletal pain who participate in an IMPT program compared to a usual care control group with a follow-up period of 12 months. Our second objective is to determine the cost-effectiveness of the AGRIPPA intervention relative to usual care. Our third objective is to explore the level of engagement and perceived usability of patients that use the app. We have formulated three hypotheses: (1) the maintenance of improvement in pain-related disability over time after IMPT will be more favorable for the enhanced treatment condition compared to usual care; (2) the effect of the app on pain disability will translate to less health care utilization and less societal costs (eg, less absenteeism), leading to a cost-effective intervention compared to treatment as usual; and (3) for participants in the enhanced treatment condition, the perceived usability, frequency of use, duration of use, and reported adherence to the AGRIPPA app will be positively associated with a favorable change over time of pain-related disability.

## Methods

### Design

We will perform a randomized controlled multicenter superiority trial with two parallel groups in the Netherlands. Both groups will receive standard IMPT, but the experimental group will be provided with the AGRIPPA app that they can access both during and after the treatment program. The nature of the intervention does not allow for masking the condition for health care providers or patients. The allocation ratio will be 1:1.

### Ethical Approval

The study activities have been reviewed and approved by the Medical Research Ethics Committee Utrecht (19/406/D). Protocol modifications that will result in significant changes of study objectives, design, or procedures will require approval by the AGRIPPA steering committee and the Medical Research Ethics Committee.

The trial will be coordinated by a senior researcher of the Lifestyle & Health Research Group of the University of Applied Sciences Utrecht. The AGRIPPA consortium that consists of all project partners (including the participating treatment centers) meets twice per year to discuss overall progress and topics such as dissemination. The AGRIPPA steering committee consists of researchers who meet four times per year to oversee the quality of the research and to decide on any substantial amendments to the initial project idea.

### Study Setting

Six treatment facilities that provide IMPT programs participate in this study (2 hospital units and 4 rehabilitation clinics). All locations provide an interdisciplinary biopsychosocial-oriented treatment program to patients within the region, leading to a mixed rural and urban population throughout the Netherlands (ie, Arnhem, Eindhoven, Hoensbroek, Roermond, Maastricht, and Wijk aan Zee).

### Eligibility Criteria

#### Patients

All patients who participate in one of the treatment programs will be eligible to participate in this study. To be admitted to one of the treatment programs, patients must be over 18 years of age and referred by a general practitioner or medical specialist for IMPT. Furthermore, patients must have received a diagnosis of chronic musculoskeletal pain (ie, pain localized in the muscles, tendons, bones, and joints) that lasts or recurs for more than 3 months, and significantly interferes with physical, psychological, and social functioning. Patients have to consent to a biopsychosocial form of treatment and to participate actively throughout the treatment program. Patients with dominant psychiatric comorbidities (eg, severe depression) and pending legal procedures that are thought to interfere with rehabilitative success will not be eligible for treatment. In all participating treatment facilities, this standard screening procedure is performed by a physician in rehabilitation medicine.

#### Health Care Providers

To qualify for participation, treatment teams will be required to attend a workshop where they will receive instruction on how to adhere to the study protocol and how to use the app. In addition, health care providers will be instructed to document notable or unexpected events and to participate in a focus group after the study period. Each treatment location is also required to provide a research assistant who is not involved with the treatment program. This research assistant will be responsible for performing the treatment allocation procedure.

### Recruitment

All patients who will start an IMPT program during the study inclusion period will be contacted by their treating physician in rehabilitation medicine and will receive a patient information letter. The patient will then have 1 to 2 weeks to consider participation and to ask any additional questions before signing the informed consent form. Instruction of the app will follow when a signed informed consent form has been provided to the research assistant. An independent research counselor will be available to all participating patients for any questions and general support during the study. Her email address will be provided via the patient information letter.

### Randomization

In participating treatment centers, treatment programs either consist of group sessions, individual sessions, or their combination. To prevent contamination, we will perform group randomization when treatment is predominantly provided in groups. When treatment is provided in individual sessions, we will randomize each patient individually using a simple randomization procedure. The randomization will be performed using random allocation cards based on computer-generated random numbers. The randomization procedure will be executed by a research assistant who is not providing treatment, and will be concealed from health care providers and patients using a set of sequentially numbered opaque and sealed envelopes. The group randomization procedure will be performed in a similar manner for each group that contains at least one participating patient.

### Interventions

#### Standard Treatment

Although there is variation in the content of each treatment program, they all adopt a similar biopsychosocial perspective on pain management [6,32]. Furthermore, each program includes (cognitive) behavioral treatment modalities (eg, graded activity, exposure in vivo, acceptance, and commitment therapy) as well as pain neuroscience education, active patient involvement, and structured team meetings to coordinate treatment and evaluate each patient’s progress. All programs are supervised by a physician in rehabilitation medicine who is responsible for patient screening, assessment, and monitoring of overall progress. The treatment team always includes a psychologist and a physical therapist or occupational therapist. At each location, patients start with pain education, where a biopsychosocial orientation toward pain will be explained. Patients also receive a manual that provides general information about the treatment program. All therapies are delivered at the location of the treatment center, except for optional workplace visits. Relapse prevention is part of regular treatment and is addressed during group treatment, individual counseling, or through specific assignments (eg, composing a relapse prevention plan).

#### Enhanced Treatment

In addition to the regular treatment condition, patients in the enhanced treatment condition will be provided access to the AGRIPPA app that is available on the Android and iOS operating systems for mobile devices. The app consists of three components: two behavioral strategies and an education module. “Insight Cards” and “Value-Based Goals” are specific behavior regulation strategies that have been derived from the SOLACE study and aim to prevent relapse. In addition, an information and education module has been developed in response to patient and health care provider preferences that were expressed during the app design phase.

##### Component 1: Insight Cards

**Figure 1:**
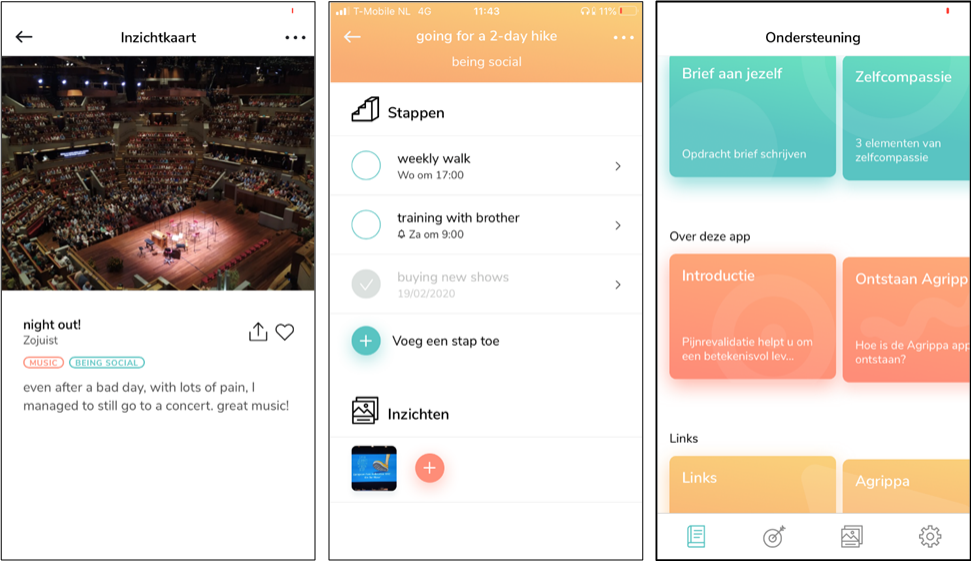
Three screenshots from the AGRIPPA app. Left: example of an insight card, including a related photo, title, two associated values, and description. Middle: Overview of a fictitious goal to go for a hike, including three steps and a related insight. Right: Overview screen of the education and information module. The first row includes treatment-specific exercises, the second row includes general information about the app, and the third row includes links to other support materials.

Patients can use “Insight Cards” to document any personally meaningful experience, thought, or idea that relates to their treatment or corresponding personal development. The main aim is that these “insights” remain accessible after treatment and thereby support the transfer of key treatment principles to each patient’s personal context. When capturing an insight, patients first type a title and a short description. Subsequently, they have the possibility to assign a corresponding value and a related picture to the insight (see Figure 1). When patients routinely save their experiences in the app during their treatment period, this will result in a chronologic overview of key experiences over time. During treatment, health care providers will be able to discuss this content with patients to check whether the treatment was received as intended. For patients, the app provides a means to reflect on important experiences during and after treatment. The app also enables patients to mark specific experiences as “favorite” and to share their Insight Cards via email or WhatsApp with their relevant others. After treatment, patients have continuous access to their personal collection of Insight Cards to recover specific insights, to explain specific insights of their condition or treatment to other people, or to reflect on their experiences. Moreover, they can add new Insight Cards to their collection after treatment completion if they have experienced relevant events or insights.

##### Component 2: Value-Based Goals

The Value-Based Goals module facilitates the formulation of meaningful goals (eg, going for a hike with friends on Saturday morning to be sociable) and subsequent action planning of each consecutive step toward this goal. The procedure is divided into four steps. First, patients formulate their overall goal. Second, patients reflect on desirability, self-efficacy, estimated time to achievement, and social support concerning this goal. Third, patients formulate a corresponding higher-order value (eg, values such as loyalty, friendship, or adventurousness in relation to a goal of going hiking with old classmates), and, optionally, a reward once the goal has been achieved. In the final part, patients can plan multiple “steps,” comprising single or recurrent activities that need to be performed to achieve the corresponding goal (eg, buying hiking shoes or planning a weekly training session). For each step, patients plan where and when the specific activity will be performed. They can also add reminders or schedule each step in their mobile calendar. In the final part of the step-planning sequence, patients reflect on potential barriers and formulate coping strategies in anticipation of these barriers (eg, preparing dinner in advance to avoid missing a training session). If a goal has been achieved, patients can directly create an Insight Card of this particular experience. Figure 1 (middle) shows an overview of steps within a specific goal.

##### Component 3: Information and Education

By default, the Information and Education module includes general information about the AGRIPPA project and instructions on how to use both strategies. In addition, each treatment center can add specific content to this section, including information materials, website links, figures, embedded videos, and assignments. The main reason for including this module is to create a single environment for all supplementary materials of the treatment program. Figure 1 (right) depicts the main screen of this module.

#### Modifications

During the training session, we recommend health care providers to regularly discuss the app during treatment. However, due to expected variations in digital literacy and other urgent topics, health care providers are allowed to modify the intensity according to their clinical judgement.

#### Adherence to the Intervention Protocol

The app will be made available to the members of the treatment teams to become accustomed to the content. The training will be provided by two researchers (JP and ES) and include an overview of the purpose and rationale of the study, as well as detailed instructions on how to use the app within the context of the treatment program. This includes identifying an appropriate moment within the treatment program to introduce the app, determining which member of the team will be responsible for the introduction and encouragement to regularly evaluate the app content, and provide feedback to the participant. During the study, a researcher will have biweekly contact with the treatment team to obtain feedback and discuss progress. Furthermore, two audit sessions will be planned at each location. The researcher will schedule an appointment to discuss overall progress, protocol adherence, and to share examples of good practice with the treatment team. During these sessions, the researcher will also inquire about substantial deviations from the protocol or discontinuation of the app during treatment.

#### Concomitant Care

During the treatment program, patients are not allowed to be treated elsewhere for their chronic pain unless the treatment team decides to refer the patient for a specific reason.

### Outcomes

**Figure 2:**
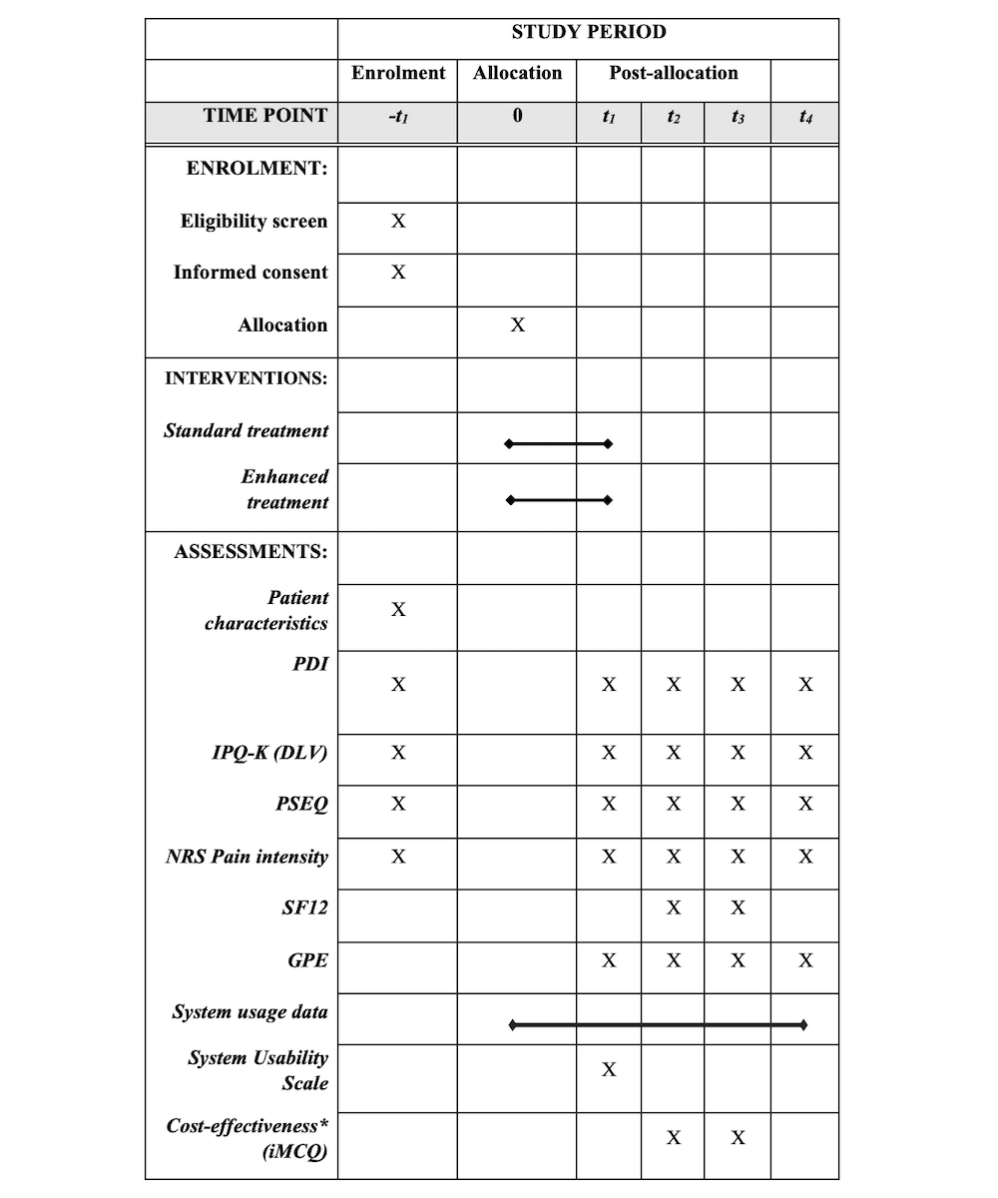
Schedule of enrollment, interventions, and assessments. Adopted from SPIRIT (2013). -t1: prior to treatment; t1: posttreatment; t2: 3 months posttreatment; t3: 6 months posttreatment; t4: 12 months posttreatment; PDI: pain disability index; IPQ-K (DLV): Dutch language version of the Illness Perception Questionnaire; PSEQ: Pain Self-Efficacy Questionnaire; NRS: numeric rating scale; SF12: 12-item short-form health survey; GPE: global perceived effect.

All six treatment facilities routinely collect outcome data with an electronic survey to monitor their patients [33]. All demographics, baseline measurements, as well as the primary outcome will be obtained by this procedure. Any outcome measures that are not part of the routine assessment will be obtained through an additional electronic survey. Table 1 includes an overview of all outcome domains, measures, and planned analysis methods. The participant flow that is depicted in Figure 2 includes all time points of data collection in this study. A researcher who is not involved with the treatment program will monitor all incoming data and will promote study retention using email reminders after 7 days and telephone reminders at 15 and 21 days postmeasurement.

**Table 1:** Planned outcome domains, outcome measures, and statistical methods.

Outcome		Hypothesis/research question	Outcome measure	Method of analysis
**Primary**				
	Self-perceived pain disability	The development of pain disability over time after IMPT^a^ will be more favorable for the enhanced treatment condition compared to usual care.	PDI^b,c^	Multilevel analysis
	Planned subgroup analysis on patients that scored 1 (“very much better”) or 2 (“much better”) on the GPE^d^ scale at *t*_1_^e^.	For patients that experienced meaningful treatment success, the development of pain disability over time after IMPT will be more favorable for the enhanced treatment condition compared to usual care.	1. PDI^c^ 2. GPE^f^	Multilevel analysis
**Secondary (quality of life)**				
	Health care costs	The enhanced treatment condition will be cost-effective compared with usual care at 3 and 6 months posttreatment.	1. SF-12^f,g^ 2. iPCQ^f,h^	Cost-effectiveness analysis
	Illness perception	The development of illness perceptions over time after IMPT will be more favorable for the enhanced treatment condition compared to usual care.	IPQ-K DLV^c,i^	Multilevel analysis
	Pain self-efficacy	The development of pain self-efficacy over time after IMPT will be more favorable for the enhanced treatment condition compared to usual care.	PSEQ^c,j^	Multilevel analysis
	Pain intensity	The development of outcome measures over time may be affected by the level of pain intensity	NRS^c,k^	Multilevel analysis
**Engagement and usability**				
	Overall engagement	The level of engagement and usability will be positively associated with a change in pain disability during follow up (*t*_4_^l^ – *t*_1_).	1. System usability scale^f^ 2. System usage data of AGRIPPA app (see below)	Multiple linear regression
	Frequency of engagement	How does the frequency of engagement vary over time after treatment?	Average number of logins per week	Descriptive statistics
	Depth of engagement	How does the average number of features accessed per login vary after treatment?	Average number of features accessed per log-in	Descriptive statistics
	Duration of engagement	How do the average minutes spent at each login as well as the total time spent with the app per week vary after treatment?	1. Average minutes spent at each login. 2. Total time spent with the app	Descriptive statistics
	Active engagement with Insight Cards	To what extent will the number of created Insight Cards increase or decrease after treatment?	Number of Insight Cards created	Descriptive statistics
	Active engagement with VBG^m^	To what extent will the number of created VBG cards increase or decrease after treatment?	1. Number of VBGs created. 2. Number of steps created	Descriptive statistics

^a^IMPT: interdisciplinary multimodal pain therapy.

^b^PDI: Pain Disability Index.

^c^Outcome measure is part of routine care.

^d^GPE: global perceived effect.

^e^*t_1_*: immediately postintervention.

^f^Outcome measures will be obtained with an additional electronic survey.

^g^SF-12: 12-item short-form health survey.

^h^iPCQ: iMTA Productivity Cost Questionnaire.

^i^IPQ-K DLV: Dutch language version of the Illness Perception Questionnaire.

^j^PSEQ: Pain Self-Efficacy Questionnaire.

^k^NRS: numeric rating scale.

^l^*t_4_*: 12 months postintervention.

^m^VBG: Value-Based Goals.

#### Primary Outcome

Self-perceived pain disability will be measured with the Pain Disability Index (PDI) [34]. This questionnaire consists of 7 items ranging from 1 (no disability) to 10 (maximum disability). Each item relates to the self-reported disability in the context of family/home, recreation, social, occupation, sexual, self-care, and life support. The PDI score (0-70) is obtained by summing all individual items. The PDI score is evaluated as responsive, with a change of 8.5 to 9.5 points considered to be clinically important [35].

#### Secondary Outcomes

##### iMTA Medical Cost Questionnaire

The iMTA Medical Cost Questionnaire (iMTQ) measures all health care costs that have been made during a specific period [36]. The questionnaire contains 18 items that refer to 11 primary care components (eg, medication use, general practitioner visits) and 5 secondary care components (eg, hospital visits). Two optional questions will be added that relate to informal care by relatives and health care–related travel expenses. Furthermore, we will add two questions from the iMTA Productivity Cost Questionnaire (iPCQ) to acquire an indication of the productivity losses, as the number of hours work lost due to the disease [37,38].

##### Twelve-Item Short Form Health Survey

The 12-item short-form health survey (SF-12) contains 12 multiple choice items that cover 8 health status domains. Raw item scores are combined and transformed into two summary scores: a physical and a mental component score [39]. Higher scores reflect better mental or physical functioning. The SF-12 is considered valid and reliable and has been previously used in patients with chronic pain [40-43].

##### Pain Self-Efficacy Questionnaire

The Pain Self-Efficacy Questionnaire (PSEQ) assesses self-efficacy beliefs regarding daily life goals in the context of chronic pain [44,45]. The questionnaire includes 10 items that ask a patient to indicate the degree of confidence to perform specific activities (eg, socializing with friends) despite the pain. Responses are obtained using a Likert scale, ranging from 0 (not confident at all) to 6 (completely confident). The sum of individual scores indicates the total pain self-efficacy. A study on the psychometric properties in a Dutch population of patients with chronic pain demonstrated that the PSEQ is an internally consistent unidimensional instrument [46].

##### Brief Illness Perceptions Questionnaire Dutch Language Versions

The Dutch language version of the Illness Perception Questionnaire (IPQ-K [DLV]) measures how patients evaluate their current health condition with respect to 8 areas of cognitive perception [47]. The questionnaire includes 9 items, with 8 items covering a different cognitive area (eg, controllability) with a Likert-scale response option, ranging from 0 (absolutely no control) to 10 (extreme amount of control). The last item requires the patient to list the 3 most important causes for their current condition. A systematic review on the clinimetric properties of the IPQ-K (DLV) concluded that the questionnaire is appropriate to explore the illness beliefs of various patient groups, including acceptable test-retest reliability. However, the smallest detectable change of 42 (on a maximum of 80 points) implies that the use of an IPQ sum score to detect individual changes is not recommended [48]. Therefore, we will not use the sum score but instead evaluate each item separately [49].

##### Pain Intensity

We will measure current pain intensity on a numerical rating scale ranging from 0 (no pain) to 10 (worst pain imaginable).

##### Global Perceived Effect

The global perceived effect (GPE) evaluates to what extent the patient’s current condition has improved or worsened compared to the period prior to the treatment program. Patients respond with a 5-point Likert scale, ranging from 1 (very much improved) to 5 (very much worsened). The psychometric properties of this questionnaire have been tested in the context of various musculoskeletal disorders and are considered adequate [50].

##### System Usability Scale

The system usability scale is a 10-item questionnaire that is frequently used for evaluation of the perceived usability of software apps [51,52]. Each item is scored on a range from strongly disagree (1) to strongly agree (5). The total usability score is expressed on a 0-100 scale with higher scores indicating more usability.

##### System Usage Data

To obtain insight into the frequency, intensity, and duration of engagement, we will obtain the following system usage data for each time point: average number of logins per week, average number of features accessed per login, average minutes spent with the app at each login, total time spent with the app per week, number of Insight Cards created, number of value-based goals created, and number of steps created within the Value-Based Goals module**.**

##### Intervention Reporting

We will ask each center to provide a detailed overview of their intervention according to the Template for Intervention Description and Replication checklist [53]. This checklist aims to provide a set of items to describe an intervention for enhancing understanding and replication. Although the interdisciplinary interventions are not the main focus of this study, we will use this intervention to provide an indication of between-center heterogeneity and to assess to what extent the interventions will be modified during the study (item 10).

### Data Management

All study data will be obtained via three electronic sources. The questionnaires will either be collected through routine monitoring procedures within the treatment centers or via additional electronic surveys. System usage data will be provided by the app developer. All study data will be stored within the firewall of the University of Applied Sciences Utrecht (UAS) in a folder on a network drive that is protected by permission rights and will only be available to researchers that are assigned by the project team to analyze the data. Data will be automatically backed up (daily) by the UAS Utrecht. To protect the identity of individual participants, we will perform the following procedures for personally identifiable information. During the data collection phase, we will pseudonymize all incoming data. In the main dataset, we will replace identity data with a unique number (ie, identifier). Date of birth will be transformed to age in years and address information will not be included in the dataset, except for province and place of residence (rural/urban). To add additional measurements to the dataset and to delete data upon participant request, we will create a correspondence table that contains both the identifier and patient personal information. This table will be stored in a separate folder that only the principal investigator (JP) and the head of the Lifestyle & Health Research Group (HW) can access. Any unforeseen data collection issues that may threaten to reveal an individual’s identity will be solved according to the recommendations of Tessier and Bonnemains [54].

### Harm

We do not expect any serious adverse events as a result of using the app. However, we will monitor any negative consequence that results from usage. During the training of the treatment team, we will instruct the health care providers to report negative experiences of the app (eg, frustration due to low digital literacy skills). During biweekly contact with treatment teams, the researcher will also actively ask for any adverse event. In the unlikely event of harm, patients can appeal to the liability insurance of the sponsor that covers any damage to research subjects caused by the study within 4 years after the end of the study.

### Data Analysis

During all analyses, the treatment condition will be masked to the researchers. We will analyze the data according to the intention-to-treat principle and include patients in the analysis regardless of their adherence to the treatment protocol. We will perform no interim analysis. We will collect data at the following time points: –t_1_ (baseline), t_1_ (immediately postintervention), t_2_ (3 months postintervention), t_3_ (6 months postintervention), and t_4_ (12 months postintervention). Prior to the main analysis, we will check the randomization by examining the distribution of baseline characteristics between both groups.

#### Sample Size Calculation

The sample size calculation is based on a 2-factor repeated-measures analysis of variance with within-factor time (5 levels) and between-factor treatment (2 levels) conditions on the outcome variable PDI. We have set the α to .05, the power (1 – β) to .95, and assumed a moderate treatment effect (*f*=0.2). To account for the expected dependencies of patients within each of the 5 participating treatment locations, we applied the Donner et al [55] formula for the variance inflation factor, assuming an intracorrelation coefficient of 0.2 [56,57]. Furthermore, we corrected the analysis for an expected attrition of 20%, which is based on the average attrition of similar studies that used the PDI [21,58,59]. Based on these calculations, a minimum sample size of 157 participants, equally divided over 5 treatment locations, will be required.

#### Primary Analysis: Pain Disability

In our primary analysis, we will test the difference in the development of pain disability over time between patients in the enhanced condition and patients in the treatment condition. To account for the assumed dependence of the repeated observations and treatment locations, we will perform a multilevel analysis. In our hierarchical model, time points (level 1) will be nested in patients (level 2) and patients nested in treatment locations (level 3). Our main analysis will include the effects of time, treatment condition, treatment location, and the interaction between time and treatment condition, with a random intercept for patients. In addition, the model will be adjusted for sex, age, pain intensity, and pain duration. In the case of a significant 2-way interaction between time and treatment condition, posthoc contrasts between the treatment conditions at 3, 6, and 12 months will be calculated. We will also perform a subgroup analysis for patients that report a positive treatment effect at t_1_ (ie, a GPE score of 1 or 2).

#### Analysis of Secondary Outcomes

##### Perceived Usability and App Engagement

Based on the results of the feasibility study and the nature of the behavior regulation strategies, we expect an engagement pattern in frequency, type, and depth of engagement that differs for each behavior change strategy and changes over time. Specifically, for Insight Cards, we believe that patients will actively engage with this component during treatment (ie, creating Insight Cards during use), but shift to more passive engagement (ie, reading the input, but only creating new content at limited occasions) during follow up. For Value-Based Goals, we expect that the formulation of goals and steps will increase during the final part of treatment, together with a growing emphasis within the treatment program on integrating newly learned strategies into daily life routines. After treatment, we anticipate that patients will engage in a reflective (eg, documenting progress) and active (ie, formulating new goals) manner with this strategy. In general, we expect a decreasing trend of the number of logins over time, but an increase in the “depth” of use (ie, the average number of features accessed per login) as well as an increase in the duration of a login. We will calculate descriptive statistics to explore patterns of engagement. Furthermore, to examine the extent to which user engagement and usability are negatively associated with the change of pain disability during follow up, we will perform a multiple regression analysis, with the change score of pain disability (t_4_ – t_1_) as the outcome variable and engagement and usability measures as predictors. We will adjust for age, sex, pain intensity, and baseline PDI.

##### Cost Effectiveness

To investigate the efficiency of the intervention, we will perform a cost-effectiveness analysis at 3 and 6 months posttreatment according to the intention-to-treat principle. We hypothesize that patients in the enhanced treatment condition will have more quality-adjusted life years (QALYs) relative to the health care expenses compared to the regular treatment condition within the 6-month study period. Expected health gain will be expressed in QALYs and calculated using the procedure of Brazier and colleagues [60] to estimate the 6-dimensional health state form (SF-6D) using the SF-12 assessment at 3 and 6 months posttreatment [39,61]. Intervention costs will be determined by the standardized cost prices for rehabilitation treatment [62]. Other health expenses will be obtained using the iMTQ at 3 and 6 months posttreatment. This questionnaire includes visits to health care providers, prescribed and over-the-counter medication, and alternative health care. We will also calculate the productivity loss due to pain-related absence from work, adopting the gross human capital approach [63]. Productivity loss will be obtained with two questions of the iPCQ at 3 and 6 months posttreatment. Total costs are calculated using the Dutch manual for cost analysis in health care research [62,64]. Following the procedure of Den Hollander and colleagues [65], a standardized cost price will be used for each hour of productivity loss. Total costs and total health gains for each condition will be used to calculate the incremental cost-effectiveness ratio (ICER). Furthermore, we will construct cost-effectiveness acceptability curves based on mean costs and using incremental costs and incremental effects, employing nonparametric bootstrapping with 5000 replications. This will result in a scatter plot over four quadrants, where each quadrant indicates a different implication for economic evaluation (ie, a combination of positive or negative costs and effects) [66].

According to the National Institute for Health Care and Excellence guidelines, all intervention costs in cost-effectiveness analyses should relate to health care or social services funding [67,68]. This excludes the development costs of the AGRIPPA app because this project has been funded with research grants. However, to account for future development and maintenance costs, we will perform a sensitivity analysis and explore various cost scenarios. We will calculate multiple ICERs, each with a different cost input value that corresponds to a possible future pricing scenario (eg, subscription, pay to download).

##### Missing Data

Following recommendations of Twisk and colleagues [69], we will perform the multilevel analysis on incomplete data, rather than using multiple imputation procedures. However, we will use the R MICE package to search for patterns of missing data across the included variables and to perform *t* tests to explore the relationships between the amount of missingness of each variable and all other variables [70].

### Participant Timeline

Patient eligibility screening, informed consent, and treatment allocation procedures, as well as the baseline assessment, will be completed prior to the start of the treatment program. Patients in the enhanced treatment condition will receive instruction on how to download and use the app, and both strategies will be explained by a member of the treatment staff. To match the existing treatment content and procedures, the moment that the app will be introduced to patients can vary between locations. During the treatment phase, the app is considered a supplement to the main treatment activities and patients are free to decide how and when to use the app. Health care providers will be instructed to encourage and facilitate using the strategies when they expect it will reinforce the treatment process. However, both strategies can largely be used independently of any treatment activity and are expected to minimally affect direct treatment time. A member of the research team will be available to the treatment staff throughout the experimental phase for additional questions, support, and discussions regarding optimal use. Following treatment, there will be no additional monitoring in the enhanced treatment condition. Patients will continue to be able to use the app at their discretion. Posttreatment data will be obtained directly posttreatment (t1), and at 3 months (t2), 6 months (t3), and 12 months (t4) posttreatment.

## Results

The trial has been registered in the Netherlands Trial Register under the identifier NL8076. The study is ongoing. The patient inclusion period started in October 2019 and is expected to end in November 2020. As of March 20, 2020, we have recruited 88 patients. Results are expected to be released in the final quarter of 2021. In the last meeting of 2020, the steering committee will initiate the formation of writing teams that will be responsible for the final trial report.

## Discussion

### Study Goals

This study will evaluate the AGRIPPA app in the context of interdisciplinary multimodal pain treatment programs. Specifically, we will investigate the effect of app use on long-term pain disability and efficiency by means of a cost-effectiveness analysis. To discover how patients interact with the app, we will also explore usability and engagement and test the impact of these variables on pain disability. Together, these analyses will help to demonstrate to what extent the AGRIPPA app contributes to preventing relapse in pain-related disability.

In contrast to prevailing intervention development guidelines, the AGRIPPA development project adopted a co-design approach and started with collecting qualitative data from end users (eg, patients and health care providers) rather than with formulating a theoretical framework. Although co-design is increasingly acknowledged in the health care domain as a method to integrate stakeholder input into the intervention design, more robust evaluations of co-design–based interventions are required to determine its additional value to existing development practices [71,72]. A similar point can be made for the evaluation of mHealth apps. A recent systematic review revealed that health care apps to promote self-management in chronic conditions have seldom been evaluated by randomized controlled trials over a prolonged study period [27]. This study will help understand if an mHealth app that has been developed by co-design methods not only contributes to an acceptable and user-friendly intervention but also leads to maintenance of treatment gains for patients with chronic pain.

### Strengths and Limitations

Because IMPT programs often substantially vary in dose and content, the inclusion of multiple treatment centers will positively influence generalization. Furthermore, this study builds on a feasibility study where evaluations related to form, content, and integration within treatment programs have been incorporated into the current app and study procedures.

The exploratory analysis of engagement variables in this study is expected to provide preliminary insight into patient adherence to the behavior regulation strategies within the app. According to Sieverink and colleagues [73], insight into adherence to mHealth apps can be acquired by combining usage behavior data with a description of intended use and a well-substantiated justification for this intended use. Although this may be difficult to quantify as the intended usage of the app depends on fluctuations of patients’ functional status in the posttreatment phase, the comparison of system usage data with our expectations regarding the use of the strategies will at least provide an indication of adherence to the app. Possible follow-up studies that include qualitative evaluations of patient input may lead to a more sustained insight into adherence.

This study includes several challenges and compromises that can potentially bias the outcomes. First, including patients with both treatment conditions within one center increases the risk of contamination. Second, health care providers have a large influence on participant engagement. Our feasibility study indicated that health care provider involvement varied greatly between patients, and that patients with limited health care provider feedback did not always use the intervention as intended (see personal communication, first author SE; manuscript under review). By scheduling regular contact moments to discuss progress, we aim to minimize the impact of this potential threat. Third, the limited project duration and funding resulted in a maximum follow-up period of 12 months. Although this first year may be crucial for integrating the newly learned management strategies into a daily life routine, the effect of the AGRIPPA app on late-onset occurrences of relapse will not be monitored in this study. Limited funding also prevented the development of an active (mHealth) control condition. Although opting for a treatment-as-usual condition as a comparator is a widely used method, this does not control for the potential placebo effect of receiving an mHealth app. Fourth, to minimize the impact for patients to participate in this study, we have selected a limited number of outcome measures and measurement time points in addition to routine assessments. This may result in an increased recall bias or limited insight in potential factors that could explain a possible effect of the app. Finally, patients will require sufficient digital literacy skills to effectively use the app, which may lead to self-selection during the recruitment phase. The selection bias may threaten generalization.

### Implications for Practice

Maintaining behavior change is notoriously difficult to achieve and every small step toward decreasing relapse or understanding the specific mechanisms by which relapse occurs or is prevented will be important to the field of pain rehabilitation. Implementation of this intervention in treatment programs may also positively empower patients to take a more proactive role in their treatment program and increase sharing their experiences, thoughts, and beliefs with health care providers and significant others. This may not only lead to a better patient–health care provider relationship and improved mutual understanding but is also expected to positively influence adherence to newly learned pain management strategies [74].

## Abbreviations

GPE: global perceived effort
ICER: incremental cost-effectiveness ratio
IMPT: interdisciplinary multimodal pain therapy
iMTQ: iMTA Medical Cost Questionnaire
iPCQ: iMTA Productivity Cost Questionnaire
IPQ-K (DLV): Illness Perception Questionnaire- Dutch language version
mHealth: mobile health
PDI: Pain Disability Index
PSEQ: Pain Self-Efficacy Questionnaire
QALYs: quality-adjusted life years
SF-6D: 6-dimensional health state form
SF-12: 12-item short-form health survey
UAS: University of Applied Sciences Utrecht
